# Circulating Levels of Interleukin-1 Family Cytokines in Overweight Adolescents

**DOI:** 10.1155/2010/958403

**Published:** 2010-02-09

**Authors:** Christian Jung, Norbert Gerdes, Michael Fritzenwanger, Hans Reiner Figulla

**Affiliations:** ^1^Department of Internal Medicine I, Friedrich-Schiller-University, Erlanger Allee 101, 07747 Jena, Germany; ^2^Department of Medicine, Center for Molecular Medicine, Karolinska Institute, 17176 Stockholm, Sweden; ^3^Institute for Molecular Cardiovascular Research (IMCAR), RWTH Aachen University, 52074 Aachen, Germany

## Abstract

*Objectives*. Obesity and related diseases are dramatically increasing problems, particularly in children and adolescents. We determined circulating levels of different interleukin (IL)-1 family members in normal weight and overweight adolescents. *Methods*. Seventy male, Caucasian adolescents (13–17 years) were recruited. Thirty-five had a body-mass index (BMI) above the 90th age-specific percentile. IL-1*α*, IL-1*β*, IL-1 receptor antagonist (IL-1ra), and IL-18 were determined using multiplex-technology. *Results*. IL-18 concentrations were higher in the overweight group compared to normal weight (161.6 ± 40.7 pg/ml versus 134.7 ± 43.4 pg/ml, *P* = .009). Concentrations of circulating IL-1*β* levels were below the detection threshold. IL-18 (*R* ^2^:0.355, *P* < .01) and IL-1ra (*R* ^2^:0.287, *P* < .05) correlated with BMI, whereas IL-1*α* did not. *Conclusions*. Accumulating data indicate the importance of the endocrine function of adipose tissue for the pathophysiological consequences of obesity-related co-morbidities. Since IL-18 is involved in the pathogenesis of different cardiovascular diseases, we conclude that IL-18 may represent a link between obesity and related co-morbidities in children and adolescents.

## 1. Introduction

Overweight and the related metabolic syndrome are dramatically increasing problems, particularly in children and adolescents [1, 2]. 

Being overweight in early life significantly increases the risk for and severity of obesity in adulthood [3]. Obesity increases the risk for related morbidities such as the metabolic syndrome (MS), which is defined as a clustering of cardiovascular risk factors, including impaired glucose tolerance, dyslipidemia, and hypertension. Obesity, especially abdominal obesity, is a hallmark of the MS and insulin resistance, a consequence of abdominal obesity. In addition, abdominal obesity is considered to be the driving force of the MS. Furthermore, type 2 diabetes mellitus (T2D) now accounts for up to 45 percent of all newly diagnosed diabetes in pediatric patients in northern America [4].

In the last decade, increasing evidence suggested the striking relevance of a low but chronic inflammatory state in obesity and MS. Population-based studies have shown strong relationship between inflammatory markers and lipid metabolism abnormalities, obesity, and resulting complications such as atherosclerosis [5, 6]. This presence of chronic low-grade inflammation in obese states, in insulin resistance, the MS, and T2D, and in early stages of atherogenesis supports the notion that inflammation may be the causal link connecting adipose tissue dysfunction with metabolic and vascular pathologies [7]. Research of recent years revealed a role of adipose tissue beyond energy storage harboring inflammatory cells which are believed to sustain inflammation and impair adipocyte function [8]. These effects might be mediated by cytokines released in substantial amounts from adipocytes, as well as from inflammatory cells recruited into adipose tissue. Many proinflammatory cytokines such as tumor necrosis factor alpha (TNF*α*) and interleukin (IL)-6, as well as other markers like leptin, adiponectin, and stromal-derived-factor (SDF) 1 in overweight children and adolescents have attracted considerable attention [9–13]. In addition, cytokines of the IL-1 family have been described to be elevated in overweight adults. This family includes among others IL-1*α*, IL-1 receptor antagonist (IL-1ra), IL-1*β*, and IL-18. Both proinflammatory cytokines, IL-1*β* and IL18, are elevated in adult obesity and share a similar signal transduction pathway [14]. The IL-1ra is an anti-inflammatory cytokine that is also produced by white adipose tissue. IL-1ra binds to the IL-1 receptor in a non-activating fashion competing with and antagonizing the proinflammatory IL-1 [15]. Systemic levels of IL-1ra were shown to be elevated in obese adults likely representing a protective response to the rise of IL-1 [16].

The aim of this study was to investigate circulating levels of IL-1 family cytokines in adolescents and the influence of obesity in early life as well as the correlation to markers of glucose metabolism (adiponectin) and endothelial damage.

## 2. Methods

### 2.1. Study Subjects

Voluntary individuals were recruited in schools of the region of Jena, Germany. Seventy male, Caucasian adolescents (aged 13–17 years) were studied. Of these 35 (50%) had a body-mass index (BMI) above the 90th percentile according to German charts [17]. Subjects and their parents gave informed consent and protocols were approved by the University's ethics committee in accordance with the Helsinki Declaration II. 

For all participants the following parameters were recorded in one consultation: age, height, weight, waist and hip circumference, heart rate, and blood pressure (systolic and diastolic). Any sign of disease was an exclusion criterion. BMI was calculated with the formula: body weight[kg]/height[cm]^2^. BMI-standard deviation score (SDS) was calculated according to current guidelines using German charts [17]. Briefly, the formula BMI-SDS= ([BMI/M(t)]^L(t)^–1)/L(t)∗S(t) was used. M(t) is the age- and sex-specific BMI median. L(t) and S(t) are age and sex-specific calculation variables available in charts.

### 2.2. Routine Laboratory

Blood samples were drawn in the morning after an overnight fast using EDTA as anticoagulant. Standard plasma parameters were obtained from the Department of Clinical Chemistry at the University Hospital Jena: high-density lipoprotein (HDL; mmol/l), low-density lipoprotein (LDL; mmol/l), triglycerides (mmol/l), and C-reactive protein (CRP, mg/l; using high sensitivity assay) were analyzed.

### 2.3. Cytokine Determination

The quantitative determination of human IL-1*α*, IL-1*β*, IL-1ra, and IL-18 was performed using immediately frozen plasma (−70°C). Cytokines were determined by Bio-Plex technology according to the manufacturer's instructions (Bio-Rad, Hercules, CA). Briefly, fluorescently-dyed microspheres coated with a capture antibody bind to relevant cytokines in a sandwich immuno-assay format. Following removal of unbound sample specific protein was detected with a fluorescently labeled antibody and analyzed in a Luminex analyzer [18].

### 2.4. ELISA

As surrogate markers for insulin sensitivity and glucose metabolism [19] and for early endothelial damage [20] and activation, adiponectin and soluble E-selectin (sE-selectin),respectively, were measured using ELISA technique according to manufacturer's instructions (R&D Systems, Wiesbaden, Germany).

### 2.5. Statistical Analysis

Cytokine levels between groups were compared with *t*-test. Correlation analyses between cytokine levels and other laboratory values or patient characteristics employed Pearson correlation coefficient. For correlation between cytokines and the ELISA markers, linear regression analysis was also used. Statistical significance was assumed if a null hypothesis could be rejected at *P* ≤ .05. All statistical analyses were performed with SPSS, version 12.0 (SPSS Inc.).

## 3. Results

The baseline characteristics are shown in Table 1. Overweight adolescents had higher BMI, BMI-SDS, waist circumference, and systolic blood pressure at rest. Levels of triglycerides, total cholesterol, and LDL did not differ, but HDL was lower in overweight adolescents. In addition, overweight adolescents had higher levels of CRP likely reflecting a low-grade inflammatory state.

Concentrations of circulating IL-1*β* levels were below the detection threshold. IL-1*α* (normal weight: 29.40 ± 7.81 pg/mL versus overweight: 31.62 ± 9.26 pg/mL; *P* = .281) and IL-1ra (normal weight: 203.79 ± 178.18 pg/mL versus overweight: 280.15 ± 213.37 pg/mL; *P* = .109) levels did not differ significantly, whereas IL-18 was higher in overweight adolescents (134.73 ± 43.38 pg/mL versus 161.57 ± 40.67 pg/mL; *P* = .009).

**Figure 1:**
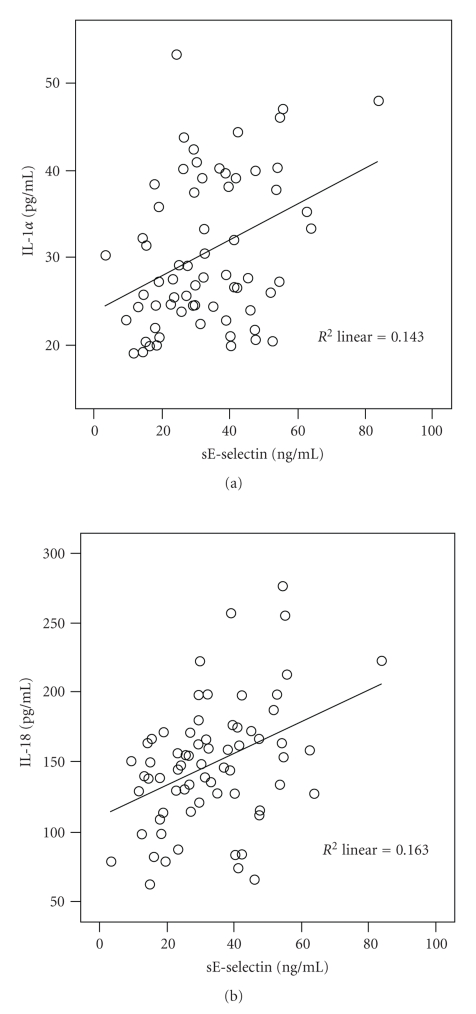
Association of soluble E-selectin (sE-selectin) with IL-1*α* (a) and IL-18 (b). Both correlations: *P* < .001.

IL-1ra and IL-18 correlated with all anthropometrical measurements of obesity, including body weight, BMI, BMI-SDS, and waist circumference, whereas IL-1*α* did not (Table 2). Blood pressure levels at rest had no association with any of the investigated cytokines. Triglycerides, total cholesterol, and LDL cholesterol correlated with IL-1*α*, whereas HDL cholesterol correlated inversely with IL-18. Higher inflammatory state determined by CRP was associated with higher IL-1*α* and IL-18. To further dissect the relevance of IL-1 family cytokines, we correlated (linear regression analysis) these markers with adiponectin as surrogate for beginning insulin resistance and glucose metabolism and sE-selectin as plasma correlate for functional and morphological changes in the vessel wall in teenagers. IL-1*α*, IL-18, and IL-1ra did not correlate with adiponectin (data not shown). Correlation of sE-selectin to IL-1ra also did not show significant results, but sE-selectin correlated to IL-1*α* and IL-18 (both *P* < .001, Figure 1).

## 4. Discussion

During the past decades, the industrialized countries witnessed a dramatic increase in the prevalence of obesity. Recent research has clearly confirmed that overweight and especially obesity are associated with a chronic subclinical inflammation [21]. Adipose tissue is not merely a simple reservoir of energy stored as triglycerides, but serves as an active secretory organ releasing many peptides and cytokines into the circulation. In the state of obesity, the balance between these numerous molecules is dysregulated. Enlargement of adipocytes producing more proinflammatory cytokines (i.e. TNF*α*, IL-6) and less anti-inflammatory proteins, such as adiponectin, was suggested as one potential explanation [22]. Others proposed that infiltration of inflammatory cells may represent the critical step in adipose tissue-associated inflammation, although the initial trigger(s) for accumulation of these cells remains elusive. The present study extends the existing knowledge about alterations in the IL-1 family in obese adults to obese teenagers.

Elevation of IL-1*α* and IL-1ra has been described under different conditions, including overweight and diabetes, while circulating levels of IL-1*β* were reported below the sensitivity of the assays [23, 24]. Of note, the pathophysiological link between obesity and resulting T2D mediated by IL-1*α*, IL-1*β*, and their antagonist IL-1ra is discussed in current literature, but is not completely understood. While IL-1*α* inhibits insulin signaling in adipocytes [25], IL-1*β* mediated cytotoxic effects in pancreatic islets consequently leading to impaired insulin secretion [26]. Therefore, IL-1ra received considerable clinical attention due to the potential therapeutic application of this natural inhibitor. Interestingly, treatment with an IL-1 antibody improves glycemic control in diet-induced obesity in mice [27]. In adolescents these cytokines—if detectable—did not differ significantly between groups. However, mean numbers in overweight adolescents showed a trend for IL-1ra to be increased and IL-1ra correlated with anthropometrical measurements of obesity, such as waist circumference, which is a good marker for visceral adipose tissue. This correlation suggests that the recruitment of this endogenous protective mechanism already starts in early life controlling activation of the IL-1 axis. Furthermore, we observed an intriguing association between IL-1*α* and sE-selectin. Invitro studies showed that IL-1*α* promotes procoagulative activity and inflammation in endothelial cells [28]. Since circulating levels of sE-selectin reflect remarkably well structural and functional changes of the vessel wall in early life [20], it is tempting to speculate whether IL-1*α* is involved into such changes. Future studies will have to elucidate this question. 

IL-18 is closely related to IL-1*β* sharing structural and functional similarities. Both the IL-1 receptor and IL-18 receptor belong to the same receptor superfamily [29]. IL-18 is implicated in the pathogenesis of several diseases including atherosclerosis and ischemic heart disease [30–32], and more recently a novel function for IL-18 in the control of energy homeostasis has also been described [33]. Zirlik et al. and others reported that circulating levels of IL-18 in human adults directly correlate with BMI, adiposity, and insulin resistance and are elevated in obesity [31, 34]. In adipose tissue, resident macrophages are the major source of IL-18 [35], supported by Fain et al., showing that the nonfat cells in human adipose tissue contribute to most of the release of IL-18 [36]. These results highlight the importance of IL-18 in obesity, furthermore, supported by animal studies showing that IL-18 modulates food-intake, leading to hyperphagia in IL-18 deficient mice [37]. In our adolescent cohort, IL-18 is elevated in overweight individuals and correlates with different anthropometrical measurements of obesity. Furthermore, it is associated with inflammatory state (CRP) and endothelial damage (sE-selectin), underlining the early development of atherosclerosis in overweight teenagers and the connecting role of inflammation mediated by factors such as IL-18. Since insulin resistance is a crucial component of MS, it is surprising that IL-18 is not associated (inverse) with adiponectin. However, our data corroborate previous studies reporting that in obese children and adults, IL-18 levels can be independent of the alterations in adiponectin [38, 39]. 

Zilverschoon et al. recently proposed a mechanism of IL-18 resistance which is of potential relevance for the understanding of the consequences of high IL-18 levels [40]. In their study obese adults are characterized by higher levels of IL-18, but their leucocytes show lower response to IL-18 stimulation. The development of IL-18 resistance may provide an explanation for the increased susceptibility of overweight subjects for infections. This hypothesis, which remains to be tested, may also hold true for children and adolescents since an increased frequency of infections was reported for obese or overweight subjects [41]. Thus, future studies have to address the correlations between IL-18 levels, obesity, and infections in early life.

We would like to discuss some strengths and weaknesses of our study: a limitation is that the study cohort is comparably small. Since IL-1ra correlated well with different anthropometrical measurements of obesity, it is reasonable to speculate that there might also be a difference between groups if group size would be bigger. However, group sizes were powerful enough to reveal striking differences for a related cytokine of the same family. An obvious strength of this study is that it—to the best of our knowledge—for the first time investigates IL-1 family cytokines in normal weight and overweight adolescents.

In summary, we conclude that cytokines of IL-1 family are involved in pathophysiological conditions that occur in overweight adolescents. Notably, IL-18 is elevated in overweight teenagers, correlating with different anthropometrical measurement of obesity and associated with sE-selectin, a marker for endothelial damage. However, IL-1*α* and IL-1ra are not significantly elevated and may not contribute to the pathophysiology that increases the frequency of diabetes in overweight adolescents. Early changes in cytokine profile underline the urgency of fighting obesity in childhood and adolescence.

## Figures and Tables

**Table 1:** Characteristics of the study population. Data are presented as mean ± standard deviation. n.s.: not significant.

	Normal weight adolescents	Overweight adolescents	*P*-value
N	35	35	
Age (Years)	15.3	15.1	n.s.
Body Weight (kg)	63.3 ± 7.6	97.0 ± 19.3	<.001
BMI	20.5 ± 1.9	32.1 ± 5.2	<.001
BMI-SDS	0.05 ± 0.68	2.39 ± 0.52	<.001
Waist circumference (cm)	72.7 ± 4.4	100.2 ± 13.5	<.001
Systolic blood pressure (mmHg)	120 ± 11	134 ± 17	.021
Triglycerides (mmol/l)	0.89 ± 0.29	1.12 ± 0.74	n.s.
Total cholesterol (mmol/l)	3.92 ± 0.79	4.09 ± 0.77	n.s.
LDL cholesterol (mmol/l)	2.37 ± 0.82	2.55 ± 0.72	n.s.
HDL cholesterol (mmol/l)	1.27 ± 0.21	1.10 ± 0.22	.002
High-sensitive CRP (mg/dl)	0.52 ± 1.49	2.69 ± 3.19	.001

**Table 2:** Correlation of IL-1*α*, IL-1ra, and IL-18 to anthropometrical measurements of obesity, patient characteristics, and different lab values. Indicated is the Pearson correlation coefficient (*R^2^*).

	IL-1*α*	IL-1ra	IL-18
Body Weight	0.217^ns^	0.366**	0.346**
BMI	0.200^ns^	0.287*	0.355**
BMI-SDS	0.198^ns^	0.284*	0.349**
Waist circumference	0.221^ns^	0.346**	0.366**
Systolic blood pressure	−0.110^ns^	−0.065^ns^	−0.302^ns^
Triglycerides	0.331**	0.242**	0.225^ns^
Total cholesterol	0.332**	0.166	0.055
LDL cholesterol	0.333**	0.120^ns^	0.202^ns^
HDL cholesterol	−0.152^ns^	−0.194^ns^	−0.239*
CRP	0.298*	0.208^ns^	0.277*

ns:not significant

*:significant at 0.05 level

**:significant at 0.01 level
